# Importance of Both Imprinted Genes and Functional Heterogeneity in Pancreatic Beta Cells: Is There a Link?

**DOI:** 10.3390/ijms22031000

**Published:** 2021-01-20

**Authors:** Pauline Chabosseau, Guy A. Rutter, Steven J. Millership

**Affiliations:** Section of Cell Biology and Functional Genomics, Division of Diabetes, Endocrinology and Metabolism, Department of Metabolism, Digestion and Reproduction, Faculty of Medicine, Imperial College London, Du Cane Road, London W12 0NN, UK; p.chabosseau@imperial.ac.uk (P.C.); g.rutter@imperial.ac.uk (G.A.R.)

**Keywords:** genomic imprinting, methylation, beta cell function, type 2 diabetes, diet, beta cell heterogeneity, pancreatic islets, single-cell transcriptomics, ‘hub’ cells, beta cell connectivity

## Abstract

Diabetes mellitus now affects more than 400 million individuals worldwide, with significant impacts on the lives of those affected and associated socio-economic costs. Although defects in insulin secretion underlie all forms of the disease, the molecular mechanisms which drive them are still poorly understood. Subsets of specialised beta cells have, in recent years, been suggested to play critical roles in “pacing” overall islet activity. The molecular nature of these cells, the means through which their identity is established and the changes which may contribute to their functional demise and “loss of influence” in both type 1 and type 2 diabetes are largely unknown. Genomic imprinting involves the selective silencing of one of the two parental alleles through DNA methylation and modified imprinted gene expression is involved in a number of diseases. Loss of expression, or loss of imprinting, can be shown in mouse models to lead to defects in beta cell function and abnormal insulin secretion. In the present review we survey the evidence that altered expression of imprinted genes contribute to loss of beta cell function, the importance of beta cell heterogeneity in normal and disease states, and hypothesise whether there is a direct link between the two.

## 1. Introduction

Adequate insulin secretion is a sine qua non for the effective control of blood glucose in mammals and defects in this process are involved in all forms of diabetes mellitus. Thus, whilst type 1 diabetes involves the immune-mediated destruction of beta cells [1], residual beta cells often remain, especially in those diagnosed later in life [2] but are unable respond adequately to stimulation with glucose. In type 2 diabetes (T2D), the beta cell mass is thought to remain mostly intact [3] but to become largely refractory to stimulation with glucose [4]. Though certain studies [5] report up to 50% beta cell “loss” in individuals with T2D, it should be emphasised that the histological studies on which these conclusions are based are not prospective (i.e., involving measurements in the same individual). Consistent with the preservation of a substantial number of beta cells, responses to non-glucose stimuli, including certain amino acids, are often largely preserved in subjects with T2D [6].

Healthy beta cells respond to glucose through the uptake of the sugar via glucose transporters (GLUT2/SLC2A2 in rodents, GLUT1-3 in man [7]), phosphorylation of the sugar by a low affinity hexokinase, termed glucokinase (encoded by the *GCK* gene [8]) and enhanced flux through glycolysis [4]. Efficient mitochondrial oxidative metabolism, achieved in part through the absence of alternative metabolic fates for glucose carbon [6,9] then drives increases in ATP/ADP ratio in the cytosol [10] which close ATP-sensitive K^+^ channels [11], depolarising the plasma membrane to facilitate Ca^2+^ entry through voltage-gated Ca^2+^ channels [4]. The latter then activate the release of stored insulin from secretory granules through regulated exocytosis. Other, less well-defined, “amplifying” glucose signalling mechanisms potentiate the actions of Ca^2+^ on the granules [12,13,14].

Compromised beta cell glucose metabolism and the misexpression of critical transcription factors and glucose sensors such as GLUT2/SLC2A2 [15,16] appear to underlie the changes which suppress normal glucose sensing in the islet in T2D. Moreover, re-expression of genes which are usually expressed at low levels (including those which are selectively “disallowed” in the beta cell, but strongly expressed in most other tissues, including *Ldha* and the lactate/pyruvate transporter MCT-1/*Slc16a1* [17]) and markers such as *Aldh1a3* [18] may also be involved in rendering beta cells “blind” to stimulation.

Heterogeneity and connectivity have emerged in recent years as important aspects of the healthy beta cell population [19] and may be compromised in both type 2 [20] and type 1 [21] diabetes. Importantly, subsets of beta cells which appear to be highly connected to other cells within a cellular network [22,23], and to influence overall islet-wide dynamics, appear to be potential targets for dysfunction in both disease settings. The molecular mechanisms leading to the establishment of an apparent beta cell “hierarchy” are poorly defined. Epigenetic changes, including alterations in DNA methylation [24], represent one possibility.

In the present review, we discuss the importance of both imprinted gene expression and functional heterogeneity in beta cells, and hypothesise that changes in imprinted gene expression may be involved in, and contribute to, the loss of beta cell function and heterogeneity in the settings of nutrient excess and T2D.

### 1.1. Genomic Imprinting

Genomic imprinting is an epigenetic phenomenon resulting in monoallelic and parent-of-origin specific gene expression [25]. The specific requirement for the individual contribution from both the male and female germlines was demonstrated in pioneering studies in the 1980s by showing that pronuclear transfer to create newly-fertilised gynogenetic (two copies of the maternal genome) or androgenetic (two copies of the paternal genome) mouse oocytes did not produce viable embryos [26,27,28,29,30]. Further studies later revealed specific regions of the genome where the presence of two maternal or two paternal chromosomal copies (known as uniparental disomy, UPD) resulted in abnormalities in early growth, development and viability, and thus formed the basis for our understanding of these so-called “imprinted regions” of the genome. The phenomenon of genomic imprinting, as well as its appearance alongside the manifestation of the placenta in mammals, have led to a number of hypotheses regarding the evolutionary advantage of “imprinting” this small subset of mammalian genes. Major examples include “parental conflict” [31] between the two genomes whereby the maternal and paternal genomes are centered around resource conservation and resource extraction, respectively, particularly during early growth and development. Co-adaptation between the mother and her offspring, stipulating that genomic imprinting may benefit their interaction, in terms of the fitness of both individuals, has also been suggested [32].

Allele-specific epigenetic control at specific imprinted *loci* is predominantly mediated by differences in methylation of cytosine residues (at the carbon-5 position) at cytosine-guanine dinucleotides or “CpGs” (reviewed extensively in [33,34]). Silencing of one parental allele is governed by an imprinting control region (ICR), that is a differentially-methylated region (DMR) often controlling multiple imprinted genes within a single genomic cluster [35,36]. The overall result is monoallelic expression of a subset of “imprinted” genes (~150 identified to date) that are defined by expression solely driven from either the paternal or maternal allele. As the epigenetic regulation at imprinted *loci* is re-established in the germline, genomic imprinting is carried through to the next generation in a transgenerational manner.

### 1.2. Human Imprinting Disorders

Imprinted genes are expressed in several metabolic systems (muscle, adipose, hypothalamic–pituitary–adrenal (HPA) axis and pancreatic beta cells), particularly at early (fetal, neonatal and postnatal) stages where they regulate a diverse range of cellular processes that ultimately mediate key physiological parameters such as growth, development, metabolism and behaviour [37]. It is, therefore, not surprising that a number of disorders exist in humans due to large chromosomal duplications (therefore leading to either paternal or maternal UPD), specific point mutations or mutations to genomic regions critical for epigenetic control of an imprinted locus (e.g., an ICR) [38,39,40,41,42]. Imprinting disorders are typically characterised by perturbed growth and development, particularly in early life, and are associated with “failure to thrive” phenotypes. Furthermore, mutant mouse lines that model human imprinting disorders generally recreate clinical features described in patients and demonstrate that even a two-fold alteration in imprinting gene expression has phenotypic consequences similar to those observed in humans [40,41]. Examples of imprinting disorders include Prader–Willi Syndrome and Angelman Syndrome, two conditions associated with major developmental and metabolic abnormalities caused by genetic disruption at 15q11-13 [43,44]. Angelman syndrome is most likely caused by disruption of the *UBE3A* gene either through direct *UBE3A* point mutations or via paternal UPD, as reviewed in [45]. Inversely, Prader–Willi Syndrome results from the deletion of paternal 15q11-13, maternal 15q11-13 UPD or ICR disruption reviewed in [46], with both disorders highlighting the importance of maintaining correct gene dosage at specific imprinted *loci*. Similar imprinting disorders have been identified with overlapping “failure to thrive” phenotypes (e.g., feeding problems, growth restriction or overgrowth, developmental delays, metabolic syndrome etc) and underlying genetic abnormalities at imprinted *loci*, for example in the cases of Silver–Russell Syndrome and Beckwith–Weidermann Syndrome (11p15.5 or “ICR2” containing *CDKN1C* and *KCNQ1*) [47,48] and Temple Syndrome (14q32.2 containing *DLK1* and *MEG3*) [49]. Importantly in the context of an impact on beta cell function and diabetes, Transient Neonatal Diabetes Mellitus (TNDM) is associated with paternal UPD of chromosome 6q24 [50,51,52,53,54] (containing the *PLAGL1*/*ZAC* gene) or modified methylation at the maternal allele [55,56] with overexpression of *Plagl1* in mice mimicking the impaired glucose homeostasis at the neonatal stage [57].

### 1.3. Imprinted Genes and Pancreatic Beta Cells

Importantly, a significant number of imprinted genes play key functional roles in beta cells, both during their early development and in the adult [58]. These include regulation of insulin secretion (*Nnat* [59,60], *Plagl1* (*ZAC1*) [57,61]), beta cell mass (*Cdkn1c* [62,63], *Dlk1* [64], *Peg3* [65], *Grb10* [66,67], *Rasgrf1* [68]) and epigenetic regulation (*Gtl2* (*MEG3*) [69,70], *H19* [71]) (Table 1). Interestingly, imprinted gene expression is deregulated in subclones of stable mouse-derived MIN6 beta cells that are “poorly responsive” in terms of insulin secretion to glucose and other secretagogues compared with “highly responsive” MIN6 subclones [72], and in pancreatic islets from T2D vs non-diabetic subjects [66,70,73,74,75]. Furthermore, single nucleotide polymorphisms (SNPs) at imprinted *loci* in multiple human cohorts are associated with T2D, possibly due to altered methylation at these genomic regions [76,77,78,79].

### 1.4. Beta Cell Heterogeneity

All beta cells are not equal, and within the same islet, individual beta cells display functional heterogeneity. Early evidence for beta cell heterogeneity was provided as long ago as 1986, using a haemolytic plaque assay developed to visualise insulin release from dispersed rat islet cells. This approach showed that beta cells are heterogeneous in terms of their ability to secrete insulin [81]. These experiments also provided evidence that cell-to-cell adhesion and/or junctional communication regulate hormone secretion from individual beta cells [81]. Other studies demonstrated intercellular differences in the secretory activity of glucose-stimulated beta cells, both in terms of glucose sensitivity and amplitude of insulin secretion. Furthermore, these highly sensitive beta cells were shown to release insulin in greater quantities at the same glucose concentration when compared to less glucose-sensitive cells [82,83,84]. Moreover, repeated stimulation with high glucose showed that individual rat beta cells from dispersed rat islets demonstrate/retain distinct and lasting secretion patterns, indicating that their excitability level remains unchanged, at least acutely [85]. Accordingly, insulin secretion from human beta cells is also heterogeneous and appears to be dependent on cell-to-cell contact [86]. Interestingly, a subset of beta cells that were poorly responsive to glucose still secreted insulin in response to other stimuli such as tolbutamide or glucagon-like peptide 1 (GLP-1) [87,88].

The molecular mechanisms that lie behind beta cell functional heterogeneity involve regulation of cellular glucose sensing and differential activity or expression of factors and pathways contributing to insulin secretion in response to glucose. For instance, variation in the expression of glucokinase (*Gck*), the flux-determining enzyme for beta cell glycolysis [4], is observed between individual beta cells from rat islets [89]. Accordingly, highly responsive beta cells have a 60% increase in glucokinase activity versus weaker responders [90]. In mouse islets expressing GFP under the control of the insulin promoter, GFP-“bright” cells (i.e., signifying a highly active insulin promoter) accounted for ~20% in comparison to GFP-“medium” cells that represented ~70% of the beta cell population [91]. Indeed, both GFP-“bright” and GFP-“medium” beta cells contained higher insulin mRNA levels and a higher secretion index when compared to GFP-“low” beta cells [91].

To further characterise beta cell heterogeneity in the human context, a study using dissociated human islets showed that four antigenically-distinct subtypes of beta cells could be identified [92]. Beta cells subpopulations designated “β1-4” display differential expression of ST8SIA1 and CD9, as well as different transcriptional signatures in general, with some of the differentially-expressed genes associated with beta cell maturation, glucose metabolism and insulin secretion [92]. These subpopulations are always present in normal adult human islets, and interestingly, the authors also described that the distribution of these beta cell subtypes is altered in T2D islets, demonstrating that beta cell heterogeneity is functionally relevant [92]. Of note, other antigenic markers have been described, such as polysialylated-neural cell adhesion molecule (PSA-NCAM) in rat beta cells, separating two populations that differ notably in their insulin secretion as well as *Gck* and *Glut2*/*Slc2a2* expression levels [93].

Another criterion of beta cell heterogeneity is whether a given subgroup has the capacity to proliferate. A study in 2016 showed that Flattop (*Fltp)*, a Wnt/planar cell polarity effector, acts as a marker gene to distinguish a subpopulation of proliferating beta cells from more mature (quiescent) beta cells, and that these two populations had distinct molecular and physiological signatures [94]. Though *Fltp* in itself is not necessary for beta cell development, proliferation or maturation, *Fltp*-positive cells showed higher insulin secretion, a lower number of immature insulin granules and higher mitochondrial function, as well as higher expression of genes involved in glucose metabolism and lower proliferation rates [94]. As shown by the several studies above, heterogeneity between beta cells can be observed at the transcriptomic level, and the impressive and rapid development of single-cell RNA sequencing technologies in recent years has allowed us to explore whole genome mRNA expression levels at cellular resolution. Despite some obvious technical limitations due to factors such as sample size, dropout effects and the requirement for efficient computational analysis methods [95,96,97], studies using single cell mRNA sequencing to explore the beta cell transcriptome have no doubt played a major role in bringing new insights for beta cells identity and heterogeneity.

The first studies that performed single cell sequencing from human pancreatic islets of healthy donors and T2D patients were stepping-stones in terms of assessing beta cell heterogeneity in the context of diabetic states and also validated previously described marker genes for endocrine cell types within the islet [98,99]. However, a limitation of these studies was the number of sequenced cells, notably beta cells. Although Li et al. explored cellular heterogeneity using a separate principal component analysis (PCA) for each cell type, the number of cells was ultimately too low to distinguish clear cellular subpopulations amongst cell types [98].

Shortly afterwards, two back-to-back studies [100,101] sequenced 2209 and 1492 single cells, respectively, from human islets of healthy and T2D donors. In addition to the large degree of overlap of gene expression for both alpha and beta cells when compared between the two studies, the number of cells sequenced also allowed Segerstolpe et al. [100] (but not Xin et al. [101]) to identify subpopulations within endocrine cell types, including beta cells. Sub-clustering of beta cells revealed five clusters with combinational expression of *RBP4*, *FFAR4*/*GPR120*, *ID1*, *ID2* and *ID3* [100] and, of note, cells of all five clusters expressed insulin (*INS*) at similar level. A later study also showed heterogeneity amongst human beta cells, in terms of the regulation of genes relating to functional maturation (*UCN3*) and ER stress (*HERPUD1*, *HSPA5* and *DDIT3*) [102]. A similar study assessed beta cell heterogeneity using an algorithm to detect outliers within the beta cell population to show that the most significant genes differentially expressed between beta cell subtypes were *SRXN1*, *SQSTM1* and three ferritin subunits, genes notably highly expressed in one of the clusters and implicated in the response to the ER and oxidative stress [103].

Very recently, Camunas-Soler et al. implemented a technique to collect both transcriptomic and electrophysiological (“Patch-seq”) data from the same endocrine cell [104]. Using human islets, the authors patch clamped 1,369 individual cells before RNA-seq analysis. This allowed them to determine how heterogeneity in gene expression correlates with functional heterogeneity recorded by patch-clamp, including exocytosis, Ca^2+^ and Na^2+^ currents. Thus, they identified genes positively or negatively associated with beta cell exocytotic capacity such as beta cell transcription factors *MAFA* and *ETV1*, insulin granule-associated *SLC30A8*, *VAMP2*, *SCG2* and *INS* as well as several metabolic enzymes and ion channels. Impressively, they also identified a gene set associated with functional heterogeneity in beta cells that can be used to predict electrophysiologal capacity [104].

Functional, metabolic, and transcriptomic heterogeneity between beta cells is now widely described in the literature (for reviews see [105,106,107,108]). A potential advantage of a heterogeneous beta cell population would be to achieve a more precise regulation of global insulin secretion while responding to different physiological conditions and, therefore, fine-tuning the control of blood glycaemia. Different excitability levels of multiple cells belonging to the same network might also contribute to the organisation of the network. For instance, one of the roles attributed to beta cell heterogeneity within the islet is to determine spatiotemporal Ca^2+^ wave dynamics in order to coordinate insulin release across the islet, where waves appear to originate in regions of the islet with elevated excitability [109]. Indeed, cells are not isolated within the islet, and islet multidimensional structure, cell-cell communication and beta cell connectivity are crucial to coordinate adequate insulin secretion in response to glucose [108]. Cell-to-cell communication is achieved by neural regulation, paracrine signalling, and possibly through primary cilia and gap junctions [20]. In mouse and human islets, beta cells are electrically coupled by Connexin 36 (*Cx36*) [110]. *Cx36*/*Gjd2* charge- and size-selective channels that notably permit intercellular passage of ions such as Ca^2+^ are important for calcium waves/oscillations, coordination and insulin secretion in the intact islet under elevated glucose [19,111,112]. Interestingly, fluorescence recovery after photobleaching (FRAP) experiments showed that cell coupling was heterogenous, with cells having either high or low coupling [113].

**Figure 1:**
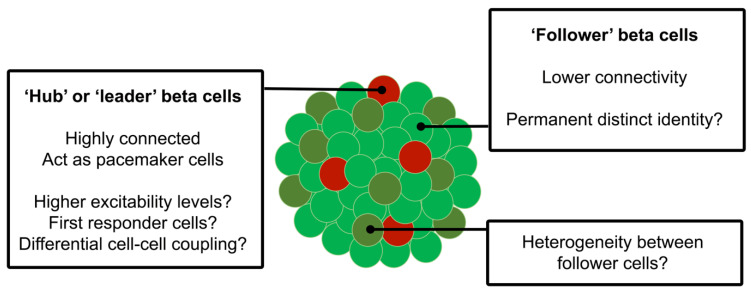
Beta cells display a heterogenous response to glucose including differences in excitability levels. A subpopulation of “hub” cells has been described that account for up to 10% of total beta cells. These “hub” cells, as opposed to “follower” beta cells, exert an acute control over islet response to high glucose and may therefore determine a key component of beta cell connectivity by coordinating the calcium response across the islet, acting as “pacemakers”.

In order to analyse beta cell connectivity, functional high-speed Ca^2+^ imaging experiments have been performed on intact islets with acquisitions subjected to computational methods to identify the cells with correlated activity; thus, a connectivity map can be constructed based on the location of significantly correlated cell pairs [20,114,115]. A subpopulation of highly-connected cells was identified in this way ex vivo, in both mouse and human islets, and these cells exerted a tight control over islet response to glucose [22]. The identified “hub” cells, which also appeared to be the first cells to show an increase in cytosolic Ca^2+^ during oscillations in this parameter and have also been termed “leaders” [116], exhibited lower PDX1 and higher GCK expression levels, as assessed by immunocytochemistry, indicative of a less mature but highly metabolic state. Hub cells accounted for ~10% of the beta cell population and, remarkably, their inactivation via an optogenetic approach in which the Cl^−^ pump halorhodopsin was activated in selected cells to achieve their reversible electrical silencing cells greatly impaired calcium dynamics across the plane of the islet interrogated. Thus, hub cells may act as “pacemaker” cells in response to elevated glucose and appear to be more sensitive than “follower” cells to pro-inflammatory factors [22] (Figure 1).

**Figure 2:**
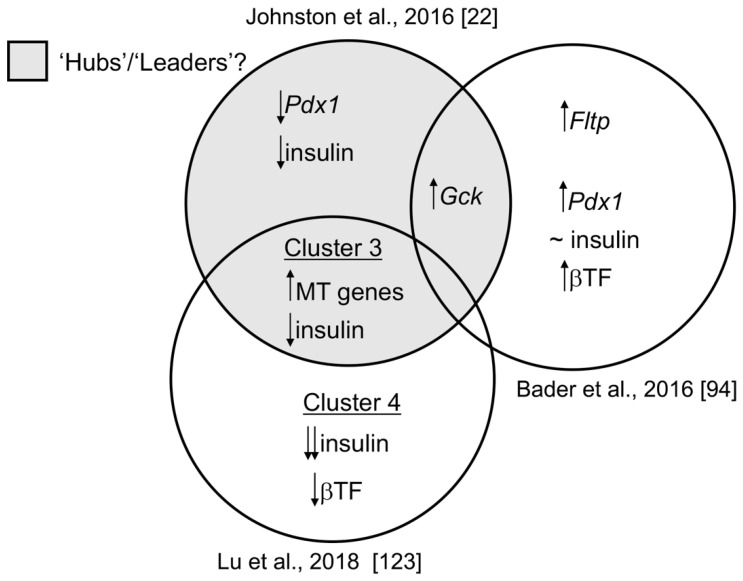
Evaluating the overlap of defined transcriptomic heterogeneity between beta cell subpopulations in mice. “Hub” cells [22] exhibit lower *Pdx1*, *Ins1* and higher *Gck* expression levels, as assessed by immunocytochemistry, indicative of a less mature but highly metabolic cell state. According to Bader et al. [94], FACS-sorting of islet beta cells into *Fltp*-positive and *Fltp*-negative populations revealed that the former was marked by expression of *Pdx1* and several key beta cell transcription factors (βTF) and, similar to “hub” cells, by increased expression of *Gck*. Expression of insulin was similar between *Fltp*-positive and *Fltp*-negative beta cells. scRNA-sequencing of primary mouse beta cells by Lu et al. [123] revealed a population of beta cells (termed “cluster 3”) with increased mitochondrial (MT) gene expression and reduced expression of insulin, but with comparable levels of beta cell transcription factors (βTF) as mature beta cells (termed “clusters 1 and 2”), consistent with “hub” cells. “Cluster 4”, in this analysis, represents immature beta cells with significantly reduced insulin levels and key beta cell transcription factors (βTF). Whether the expression of imprinted genes is enriched in any of the above subpopulations remains to be determined. Black arrows represent up- or down regulation of gene expression.

Hub cells were also observed in mouse islets as well as in the living fish embryo, with the former becoming revascularized and innervated when engrafted into the anterior chamber of the eye [116]. Similar to what was observed in mouse islets in vitro, photo-ablation of “leader” cells in the zebrafish led to loss of a coordinated calcium response [116], confirming their possible role as pacemakers. Moreover, these new findings suggest that beta cell “hubbiness” is an intrinsic property of this population and not simply reflective of the localisation of these cells within the islet (e.g., their proximity to blood vessels, nerve termini, etc.). Others [117] have demonstrated that optogenetic activation of subpopulations leads to the activation of Ca^2+^ waves, consistent with the above model, though the degree to which different sub-groups of “hubs”, “leaders” and “first responders” overlap is a matter of contention. Importantly, the means through which “hubs” transmit Ca^2+^ waves across the islet remains unclear, with both a direct mechanism involving cell-cell contacts and gap junctions [118] and the involvement of other cell types such as delta cells [119] both possible. Indeed, whilst theoretical considerations have prompted some authors to query the role of gap junctions [120], the grounds for these concerns can be questioned [121], and modelling by others [122] is consistent with these cells playing a coordinating role though gap junctions. Certain characteristics of this “hub/leader” subpopulation are nevertheless still unclear, including their complete transcriptomic (and proteomic) signature. Nevertheless, analysis of published RNA-seq data has shown higher *Gck* and lower *Pdx1* and insulin expression, and revealed a notable enrichment in genes involved in glucose oxidation [116]. We attempt, in Figure 2, to ascribe known differences in gene expression in the mouse to different beta cell subclusters [22,94,123]. Whether imprinted gene expression differs between beta cell populations (e.g., hubs and followers) is yet to be established and is an active area of research.

### 1.5. Transcriptomic Diversity between Beta Cell Subpopulations

Early work [81,83,84] and recent single cell transcriptomic profiling [92,99] and imaging studies [22,94,116] have all demonstrated functional heterogeneity amongst individual beta cells within the islet in terms of metabolism, Ca^2+^ influx and insulin secretion. These studies have also revealed diverse transcriptional signatures and secretory profiles amongst beta cell subpopulations, and alterations in subtype distribution in T2D that are associated with partial dedifferentiation and loss of beta cell “identity” [92,99]. Cells from the ‘hub’ beta cell subpopulation described above [22,116], appear to be transcriptionally immature and highly metabolic. Targeting of these hubs by “glucolipotoxic” insults may thus contribute to the development of T2D [22,116].

**Figure 3:**
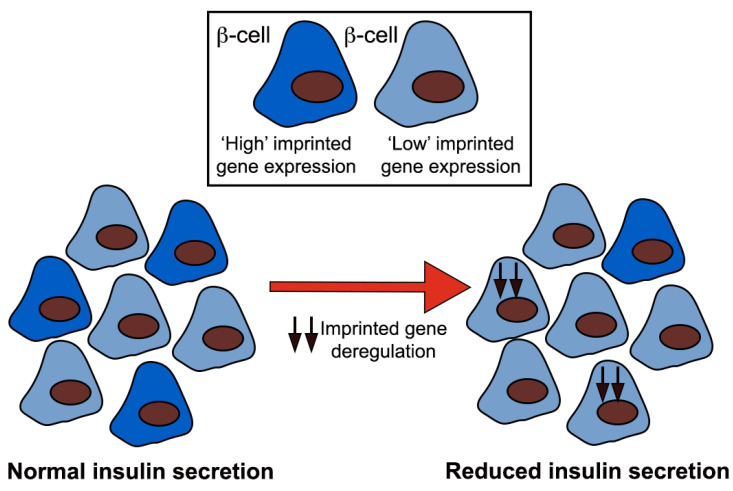
A proposed model for beta cell heterogeneity in terms of imprinted gene expression. Deregulation of imprinted gene expression by environmental factors (i.e., diet) would, in this scenario, reduce beta cell heterogeneity and therefore insulin secretion. Black arrows represent deregulation of imprinted gene expression.

Imprinted genes play key functional roles in beta cells [57,59,60,61,62,63,64,65,66,67,69,70,71] and a disproportionate number display deregulated expression in a model of diminished glucose-stimulated insulin secretion (GSIS) [72] and in pancreatic islets from T2D patients [66,70,73,74,75]. Interestingly, overnutrition (high fat or high sugar diets) has been linked to long-term, programmed epigenetic changes in gene expression at imprinted *loci* in humans and rodents [124,125,126]. It will be interesting to investigate whether imprinted genes are preferentially expressed across beta cell subtypes and whether or not differences between expression of these genes in different subsets impact beta cell heterogeneity and islet function in both normal and diabetic states (Figure 3). In this scenario, the targeting of imprinted genes, in *loci* with well understood epigenetic control and functional importance in beta cells, would enable us to understand the type and genomic distribution of epigenetic and transcriptional control that mediates stable gene expression between beta cell subtypes and their modification by environmental factors [127] (e.g., diet).

Conclusions: a role for altered imprinted gene expression in reducing beta cell heterogeneity and function?

As discussed above, alterations in the islet transcriptome are likely to be a key driver of beta cell dysfunction in diabetes. Important questions for the future are whether imprinted genes are mis-expressed in beta cells in models of type 1 and type 2 diabetes and whether such altered expression is driven by epigenetic pathways that are key to controlling imprinted gene expression (DNA methylation, modifications to histone proteins). New tools, including those in which imprinted *loci* can be examined in mice through the “knock-in” of reporter genes such as firefly luciferase [128,129], may provide an exciting means to determine the extent to which gene dysregulation occurs over time in the beta cell in the living animal and whether these changes are reversible. Imprinted genes, with their transgenerational epigenetic maintenance and functional importance in pancreatic beta cells, therefore provide an excellent opportunity to assess epigenetic change in the context of overnutrition and in other settings such as gestational diabetes, with previous studies also linking the possibility of altered imprinted gene expression and perturbed beta cell function being “passed on” from diabetic parents to the next generation [125,126,130,131,132].

## Figures and Tables

**Table 1:** Effect of modified imprinted gene expression on the function of mouse and human pancreatic beta cells.

Imprinted Gene	Proposed Functional Role in Beta Cells	Effect of Altered Expression in Beta Cells	References
*Cdkn1c*	Cell cycle control	Increased beta cell replication upon knockdown in human islets	[62,63]
*Dlk1*	Cellular differentiation	Overexpression resulted in differentiation of human pancreatic ductal cells into beta-like cells and an increase in insulin secretion	[64]
*Grb10*	Receptor tyrosine kinase adaptor protein	Knockdown in human islets reduced insulin secretion. However, increased beta cell mass, insulin secretion and improved whole body glucose tolerance in knockout mice	[66,67,80]
*Gtl2*	Long non-coding RNA	Knockdown in stable mouse beta cells increased sensitivity to cytokine-mediated oxidative stress	[70]
*H19*	Long non-coding RNA	Knockdown decreased rat beta cell expansion	[71]
*Nnat*	Mediator of preproinsulin processing	Knockout in mice leads to reduced beta cell insulin content, glucose-stimulated insulin secretion (GSIS) and glucose tolerance	[59,60]
*Peg3*	Zinc finger protein, regulates apoptosis	Viral-mediated knockdown in vitro activates beta cell cycling	[65]
*Plagl1*	Zinc finger protein, suppresses cell growth	Transient neonatal diabetes upon overexpression of *Plagl1* in mice	[52,57]
*Rasgrf1*	Guanine nucleotide exchange factor	Knockout in mice leads to reduced beta cell mass, hypoinsulinaemia and impaired glucose tolerance	[68]

## References

[1] Bluestone J.A., Herold K., Eisenbarth G. (2010). Genetics, pathogenesis and clinical interventions in type 1 diabetes. Nature.

[2] Leete P., Willcox A., Krogvold L., Dahl-Jorgensen K., Foulis A.K., Richardson S.J., Morgan N.G. (2016). Differential Insulitic Profiles Determine the Extent of beta-Cell Destruction and the Age at Onset of Type 1 Diabetes. Diabetes.

[3] Rahier J., Guiot Y., Goebbels R.M., Sempoux C., Henquin J.C. (2008). Pancreatic beta-cell mass in European subjects with type 2 diabetes. Diabetes Obes. Metab..

[4] Rutter G.A., Pullen T.J., Hodson D.J., Martinez-Sanchez A. (2015). Pancreatic beta-cell identity, glucose sensing and the control of insulin secretion. Biochem. J..

[5] Butler A.E., Janson J., Soeller W.C., Butler P.C. (2003). Increased beta-cell apoptosis prevents adaptive increase in beta-cell mass in mouse model of type 2 diabetes: Evidence for role of islet amyloid formation rather than direct action of amyloid. Diabetes.

[6] Rutter G.A., Georgiadou E., Martinez-Sanchez A., Pullen T.J. (2020). Metabolic and functional specialisations of the pancreatic beta cell: Gene disallowance, mitochondrial metabolism and intercellular connectivity. Diabetologia.

[7] Thorens B. (2015). GLUT2, glucose sensing and glucose homeostasis. Diabetologia.

[8] Iynedjian P.B. (2009). Molecular physiology of mammalian glucokinase. Cell Mol. Life Sci..

[9] Sekine N., Cirulli V., Regazzi R., Brown L.J., Gine E., Tamarit-Rodriguez J., Girotti M., Marie S., MacDonald M.J., Wollheim C.B. (1994). Low lactate dehydrogenase and high mitochondrial glycerol phosphate dehydrogenase in pancreatic beta-cells. Potential role in nutrient sensing. J. Biol. Chem..

[10] Tarasov A.I., Semplici F., Ravier M.A., Bellomo E.A., Pullen T.J., Gilon P., Sekler I., Rizzuto R., Rutter G.A. (2012). The mitochondrial Ca^2+^ uniporter MCU is essential for glucose-induced ATP increases in pancreatic beta-cells. PLoS ONE.

[11] Rorsman P., Ashcroft F.M. (2018). Pancreatic beta-Cell Electrical Activity and Insulin Secretion: Of Mice and Men. Physiol. Rev..

[12] Henquin J.C. (2011). The dual control of insulin secretion by glucose involves triggering and amplifying pathways in beta-cells. Diabetes Res. Clin. Pract..

[13] Ferdaoussi M., Dai X., Jensen M.V., Wang R., Peterson B.S., Huang C., Ilkayeva O., Smith N., Miller N., Hajmrle C. (2015). Isocitrate-to-SENP1 signaling amplifies insulin secretion and rescues dysfunctional beta cells. J. Clin. Investig..

[14] Prentki M., Corkey B.E., Madiraju S.R.M. (2020). Lipid-associated metabolic signalling networks in pancreatic beta cell function. Diabetologia.

[15] Del Guerra S., Lupi R., Marselli L., Masini M., Bugliani M., Sbrana S., Torri S., Pollera M., Boggi U., Mosca F. (2005). Functional and molecular defects of pancreatic islets in human type 2 diabetes. Diabetes.

[16] Hunter C.S., Stein R.W. (2017). Evidence for Loss in Identity, De-Differentiation, and Trans-Differentiation of Islet beta-Cells in Type 2 Diabetes. Front. Genet..

[17] Pullen T.J., Huising M.O., Rutter G.A. (2017). Analysis of Purified Pancreatic Islet Beta and Alpha Cell Transcriptomes Reveals 11beta-Hydroxysteroid Dehydrogenase (Hsd11b1) as a Novel Disallowed Gene. Front. Genet..

[18] Ishida E., Kim-Muller J.Y., Accili D. (2017). Pair Feeding, but Not Insulin, Phloridzin, or Rosiglitazone Treatment, Curtails Markers of beta-Cell Dedifferentiation in db/db Mice. Diabetes.

[19] Benninger R.K., Zhang M., Head W.S., Satin L.S., Piston D.W. (2008). Gap junction coupling and calcium waves in the pancreatic islet. Biophys. J..

[20] Rutter G.A., Hodson D.J. (2015). Beta cell connectivity in pancreatic islets: A type 2 diabetes target?. Cell Mol. Life Sci..

[21] Benninger R.K.P., Dorrell C., Hodson D.J., Rutter G.A. (2018). The Impact of Pancreatic Beta Cell Heterogeneity on Type 1 Diabetes Pathogenesis. Curr. Diab. Rep..

[22] Johnston N.R., Mitchell R.K., Haythorne E., Pessoa M.P., Semplici F., Ferrer J., Piemonti L., Marchetti P., Bugliani M., Bosco D. (2016). Beta Cell Hubs Dictate Pancreatic Islet Responses to Glucose. Cell Metab..

[23] Gosak M., Stozer A., Markovic R., Dolensek J., Perc M., Rupnik M.S., Marhl M. (2017). Critical and Supercritical Spatiotemporal Calcium Dynamics in Beta Cells. Front. Physiol..

[24] Dhawan S., Tschen S.I., Zeng C., Guo T., Hebrok M., Matveyenko A., Bhushan A. (2015). DNA methylation directs functional maturation of pancreatic beta cells. J. Clin. Investig..

[25] Surani M.A. (1994). Genomic imprinting: Control of gene expression by epigenetic inheritance. Curr. Opin. Cell. Biol..

[26] Surani M.A., Barton S.C. (1983). Development of gynogenetic eggs in the mouse: Implications for parthenogenetic embryos. Science.

[27] Surani M.A., Barton S.C., Norris M.L. (1984). Development of reconstituted mouse eggs suggests imprinting of the genome during gametogenesis. Nature.

[28] McGrath J., Solter D. (1983). Nuclear transplantation in mouse embryos. J. Exp. Zool..

[29] McGrath J., Solter D. (1983). Nuclear transplantation in the mouse embryo by microsurgery and cell fusion. Science.

[30] McGrath J., Solter D. (1984). Completion of mouse embryogenesis requires both the maternal and paternal genomes. Cell.

[31] Moore T., Haig D. (1991). Genomic imprinting in mammalian development: A parental tug-of-war. Trends Genet..

[32] Wolf J.B., Hager R. (2006). A maternal-offspring coadaptation theory for the evolution of genomic imprinting. PLoS Biol..

[33] Bartolomei M.S., Ferguson-Smith A.C. (2011). Mammalian genomic imprinting. Cold Spring Harb. Perspect. Biol..

[34] Tucci V., Isles A.R., Kelsey G., Ferguson-Smith A.C., Erice Imprinting G. (2019). Genomic Imprinting and Physiological Processes in Mammals. Cell.

[35] Ferguson-Smith A.C. (2011). Genomic imprinting: The emergence of an epigenetic paradigm. Nat. Rev. Genet..

[36] Reik W., Walter J. (2001). Genomic imprinting: Parental influence on the genome. Nat. Rev. Genet..

[37] Radford E.J., Ferron S.R., Ferguson-Smith A.C. (2011). Genomic imprinting as an adaptative model of developmental plasticity. FEBS Lett..

[38] Rampersaud E., Mitchell B.D., Naj A.C., Pollin T.I. (2008). Investigating parent of origin effects in studies of type 2 diabetes and obesity. Curr. Diabetes Rev..

[39] Eggermann T., Perez de Nanclares G., Maher E.R., Temple I.K., Tumer Z., Monk D., Mackay D.J., Gronskov K., Riccio A., Linglart A. (2015). Imprinting disorders: A group of congenital disorders with overlapping patterns of molecular changes affecting imprinted loci. Clin. Epigenetics.

[40] Peters J. (2014). The role of genomic imprinting in biology and disease: An expanding view. Nat. Rev. Genet..

[41] Soellner L., Begemann M., Mackay D.J., Gronskov K., Tumer Z., Maher E.R., Temple I.K., Monk D., Riccio A., Linglart A. (2017). Recent Advances in Imprinting Disorders. Clin. Genet..

[42] Matsubara K., Kagami M., Fukami M. (2018). Uniparental disomy as a cause of pediatric endocrine disorders. Clin. Pediatr. Endocrinol..

[43] Relkovic D., Doe C.M., Humby T., Johnstone K.A., Resnick J.L., Holland A.J., Hagan J.J., Wilkinson L.S., Isles A.R. (2010). Behavioural and cognitive abnormalities in an imprinting centre deletion mouse model for Prader-Willi syndrome. Eur. J. Neurosci..

[44] Gurrieri F., Accadia M. (2009). Genetic imprinting: The paradigm of Prader-Willi and Angelman syndromes. Endocr. Dev..

[45] Chamberlain S.J., Lalande M. (2010). Angelman syndrome, a genomic imprinting disorder of the brain. J. Neurosci..

[46] Butler M.G. (2011). Prader-Willi Syndrome: Obesity due to Genomic Imprinting. Curr. Genom..

[47] Chiesa N., De Crescenzo A., Mishra K., Perone L., Carella M., Palumbo O., Mussa A., Sparago A., Cerrato F., Russo S. (2012). The KCNQ1OT1 imprinting control region and non-coding RNA: New properties derived from the study of Beckwith-Wiedemann syndrome and Silver-Russell syndrome cases. Hum. Mol. Genet..

[48] Netchine I., Rossignol S., Azzi S., Brioude F., Le Bouc Y. (2012). Imprinted anomalies in fetal and childhood growth disorders: The model of Russell-Silver and Beckwith-Wiedemann syndromes. Endocr. Dev..

[49] Ioannides Y., Lokulo-Sodipe K., Mackay D.J., Davies J.H., Temple I.K. (2014). Temple syndrome: Improving the recognition of an underdiagnosed chromosome 14 imprinting disorder: An analysis of 51 published cases. J. Med. Genet..

[50] Temple I.K., Gardner R.J., Robinson D.O., Kibirige M.S., Ferguson A.W., Baum J.D., Barber J.C., James R.S., Shield J.P. (1996). Further evidence for an imprinted gene for neonatal diabetes localised to chromosome 6q22-q23. Hum. Mol. Genet..

[51] Temple I.K., James R.S., Crolla J.A., Sitch F.L., Jacobs P.A., Howell W.M., Betts P., Baum J.D., Shield J.P. (1995). An imprinted gene(s) for diabetes?. Nat. Genet..

[52] Gardner R.J., Mackay D.J., Mungall A.J., Polychronakos C., Siebert R., Shield J.P., Temple I.K., Robinson D.O. (2000). An imprinted locus associated with transient neonatal diabetes mellitus. Hum. Mol. Genet..

[53] Mitchell B.D., Pollin T.I. (2010). Genomic imprinting in diabetes. Genome Med..

[54] Mackay D.J., Temple I.K. (2010). Transient neonatal diabetes mellitus type 1. Am. J. Med. Genet. C Semin. Med. Genet..

[55] Kamiya M., Judson H., Okazaki Y., Kusakabe M., Muramatsu M., Takada S., Takagi N., Arima T., Wake N., Kamimura K. (2000). The cell cycle control gene ZAC/PLAGL1 is imprinted—A strong candidate gene for transient neonatal diabetes. Hum. Mol. Genet..

[56] Varrault A., Bilanges B., Mackay D.J., Basyuk E., Ahr B., Fernandez C., Robinson D.O., Bockaert J., Journot L. (2001). Characterization of the methylation-sensitive promoter of the imprinted ZAC gene supports its role in transient neonatal diabetes mellitus. J. Biol. Chem..

[57] Ma D., Shield J.P., Dean W., Leclerc I., Knauf C., Burcelin R.R., Rutter G.A., Kelsey G. (2004). Impaired glucose homeostasis in transgenic mice expressing the human transient neonatal diabetes mellitus locus, TNDM. J. Clin. Investig..

[58] Millership S.J., Van de Pette M., Withers D.J. (2019). Genomic imprinting and its effects on postnatal growth and adult metabolism. Cell Mol. Life Sci..

[59] Millership S.J., Da Silva Xavier G., Choudhury A.I., Bertazzo S., Chabosseau P., Pedroni S.M., Irvine E.E., Montoya A., Faull P., Taylor W.R. (2018). Neuronatin regulates pancreatic beta cell insulin content and secretion. J. Clin. Investig..

[60] Joe M.K., Lee H.J., Suh Y.H., Han K.L., Lim J.H., Song J., Seong J.K., Jung M.H. (2008). Crucial roles of neuronatin in insulin secretion and high glucose-induced apoptosis in pancreatic beta-cells. Cell. Signal..

[61] Hoffmann A., Spengler D. (2012). Transient neonatal diabetes mellitus gene Zac1 impairs insulin secretion in mice through Rasgrf1. Mol. Cell. Biol..

[62] Avrahami D., Li C., Yu M., Jiao Y., Zhang J., Naji A., Ziaie S., Glaser B., Kaestner K.H. (2014). Targeting the cell cycle inhibitor p57Kip2 promotes adult human beta cell replication. J. Clin. Investig..

[63] Ou K., Yu M., Moss N.G., Wang Y.J., Wang A.W., Nguyen S.C., Jiang C., Feleke E., Kameswaran V., Joyce E.F. (2019). Targeted demethylation at the CDKN1C/p57 locus induces human beta cell replication. J. Clin. Investig..

[64] Rhee M., Lee S.H., Kim J.W., Ham D.S., Park H.S., Yang H.K., Shin J.Y., Cho J.H., Kim Y.B., Youn B.S. (2016). Preadipocyte factor 1 induces pancreatic ductal cell differentiation into insulin-producing cells. Sci. Rep..

[65] Sojoodi M., Stradiot L., Tanaka K., Heremans Y., Leuckx G., Besson V., Staels W., Van de Casteele M., Marazzi G., Sassoon D. (2016). The zinc finger transcription factor PW1/PEG3 restrains murine beta cell cycling. Diabetologia.

[66] Prokopenko I., Poon W., Magi R., Prasad B.R., Salehi S.A., Almgren P., Osmark P., Bouatia-Naji N., Wierup N., Fall T. (2014). A central role for GRB10 in regulation of islet function in man. PLoS Genet..

[67] Zhang J., Zhang N., Liu M., Li X., Zhou L., Huang W., Xu Z., Liu J., Musi N., DeFronzo R.A. (2012). Disruption of growth factor receptor-binding protein 10 in the pancreas enhances beta-cell proliferation and protects mice from streptozotocin-induced beta-cell apoptosis. Diabetes.

[68] Font de Mora J., Esteban L.M., Burks D.J., Nunez A., Garces C., Garcia-Barrado M.J., Iglesias-Osma M.C., Moratinos J., Ward J.M., Santos E. (2003). Ras-GRF1 signaling is required for normal beta-cell development and glucose homeostasis. EMBO J..

[69] Wang N., Zhu Y., Xie M., Wang L., Jin F., Li Y., Yuan Q., De W. (2018). Long Noncoding RNA Meg3 Regulates Mafa Expression in Mouse Beta Cells by Inactivating Rad21, Smc3 or Sin3alpha. Cell Physiol. Biochem..

[70] Kameswaran V., Golson M.L., Ramos-Rodriguez M., Ou K., Wang Y.J., Zhang J., Pasquali L., Kaestner K.H. (2018). The Dysregulation of the DLK1-MEG3 Locus in Islets from Patients With Type 2 Diabetes is Mimicked by Targeted Epimutation of Its Promoter with TALE-DNMT Constructs. Diabetes.

[71] Sanchez-Parra C., Jacovetti C., Dumortier O., Lee K., Peyot M.L., Guay C., Prentki M., Laybutt D.R., Van Obberghen E., Regazzi R. (2018). Contribution of the Long Noncoding RNA H19 to beta-Cell Mass Expansion in Neonatal and Adult Rodents. Diabetes.

[72] Yamato E., Tashiro F., Miyazaki J. (2013). Microarray analysis of novel candidate genes responsible for glucose-stimulated insulin secretion in mouse pancreatic beta cell line MIN6. PLoS ONE.

[73] Fadista J., Vikman P., Laakso E.O., Mollet I.G., Esguerra J.L., Taneera J., Storm P., Osmark P., Ladenvall C., Prasad R.B. (2014). Global genomic and transcriptomic analysis of human pancreatic islets reveals novel genes influencing glucose metabolism. Proc. Natl. Acad. Sci. USA.

[74] Lawlor N., George J., Bolisetty M., Kursawe R., Sun L., Sivakamasundari V., Kycia I., Robson P., Stitzel M.L. (2017). Single-cell transcriptomes identify human islet cell signatures and reveal cell-type-specific expression changes in type 2 diabetes. Genome Res..

[75] Dayeh T., Volkov P., Salo S., Hall E., Nilsson E., Olsson A.H., Kirkpatrick C.L., Wollheim C.B., Eliasson L., Ronn T. (2014). Genome-wide DNA methylation analysis of human pancreatic islets from type 2 diabetic and non-diabetic donors identifies candidate genes that influence insulin secretion. PLoS Genet..

[76] Kong A., Steinthorsdottir V., Masson G., Thorleifsson G., Sulem P., Besenbacher S., Jonasdottir A., Sigurdsson A., Kristinsson K.T., Jonasdottir A. (2009). Parental origin of sequence variants associated with complex diseases. Nature.

[77] Yasuda K., Miyake K., Horikawa Y., Hara K., Osawa H., Furuta H., Hirota Y., Mori H., Jonsson A., Sato Y. (2008). Variants in KCNQ1 are associated with susceptibility to type 2 diabetes mellitus. Nat. Genet..

[78] Unoki H., Takahashi A., Kawaguchi T., Hara K., Horikoshi M., Andersen G., Ng D.P., Holmkvist J., Borch-Johnsen K., Jorgensen T. (2008). SNPs in KCNQ1 are associated with susceptibility to type 2 diabetes in East Asian and European populations. Nat. Genet..

[79] Rampersaud E., Damcott C.M., Fu M., Shen H., McArdle P., Shi X., Shelton J., Yin J., Chang Y.P., Ott S.H. (2007). Identification of novel candidate genes for type 2 diabetes from a genome-wide association scan in the Old Order Amish: Evidence for replication from diabetes-related quantitative traits and from independent populations. Diabetes.

[80] Smith F.M., Holt L.J., Garfield A.S., Charalambous M., Koumanov F., Perry M., Bazzani R., Sheardown S.A., Hegarty B.D., Lyons R.J. (2007). Mice with a disruption of the imprinted Grb10 gene exhibit altered body composition, glucose homeostasis, and insulin signaling during postnatal life. Mol. Cell. Biol..

[81] Salomon D., Meda P. (1986). Heterogeneity and contact-dependent regulation of hormone secretion by individual B cells. Exp. Cell Res..

[82] Bosco D., Meda P. (1991). Actively synthesizing beta-cells secrete preferentially after glucose stimulation. Endocrinology.

[83] Kiekens R., In’t Veld P., Mahler T., Schuit F., Van De Winkel M., Pipeleers D. (1992). Differences in glucose recognition by individual rat pancreatic B cells are associated with intercellular differences in glucose-induced biosynthetic activity. J. Clin. Investig..

[84] Van Schravendijk C.F., Kiekens R., Pipeleers D.G. (1992). Pancreatic beta cell heterogeneity in glucose-induced insulin secretion. J. Biol. Chem..

[85] Giordano E., Bosco D., Cirulli V., Meda P. (1991). Repeated glucose stimulation reveals distinct and lasting secretion patterns of individual rat pancreatic B cells. J. Clin. Investig..

[86] Wojtusciszyn A., Armanet M., Morel P., Berney T., Bosco D. (2008). Insulin secretion from human beta cells is heterogeneous and dependent on cell-to-cell contacts. Diabetologia.

[87] Soria B., Chanson M., Giordano E., Bosco D., Meda P. (1991). Ion channels of glucose-responsive and -unresponsive beta-cells. Diabetes.

[88] Holz G.G.t., Kuhtreiber W.M., Habener J.F. (1993). Pancreatic beta-cells are rendered glucose-competent by the insulinotropic hormone glucagon-like peptide-1(7-37). Nature.

[89] Jetton T.L., Magnuson M.A. (1992). Heterogeneous expression of glucokinase among pancreatic beta cells. Proc. Natl. Acad. Sci. USA.

[90] Heimberg H., De Vos A., Vandercammen A., Van Schaftingen E., Pipeleers D., Schuit F. (1993). Heterogeneity in glucose sensitivity among pancreatic beta-cells is correlated to differences in glucose phosphorylation rather than glucose transport. EMBO J..

[91] Katsuta H., Aguayo-Mazzucato C., Katsuta R., Akashi T., Hollister-Lock J., Sharma A.J., Bonner-Weir S., Weir G.C. (2012). Subpopulations of GFP-marked mouse pancreatic beta-cells differ in size, granularity, and insulin secretion. Endocrinology.

[92] Dorrell C., Schug J., Canaday P.S., Russ H.A., Tarlow B.D., Grompe M.T., Horton T., Hebrok M., Streeter P.R., Kaestner K.H. (2016). Human islets contain four distinct subtypes of beta cells. Nat. Commun..

[93] Karaca M., Castel J., Tourrel-Cuzin C., Brun M., Geant A., Dubois M., Catesson S., Rodriguez M., Luquet S., Cattan P. (2009). Exploring functional beta-cell heterogeneity in vivo using PSA-NCAM as a specific marker. PLoS ONE.

[94] Bader E., Migliorini A., Gegg M., Moruzzi N., Gerdes J., Roscioni S.S., Bakhti M., Brandl E., Irmler M., Beckers J. (2016). Identification of proliferative and mature beta-cells in the islets of Langerhans. Nature.

[95] Prasad R.B., Groop L. (2016). Single-Cell Sequencing of Human Pancreatic Islets-New Kids on the Block. Cell Metab..

[96] Carrano A.C., Mulas F., Zeng C., Sander M. (2017). Interrogating islets in health and disease with single-cell technologies. Mol. Metab..

[97] Wang Y.J., Kaestner K.H. (2019). Single-Cell RNA-Seq of the Pancreatic Islets—A Promise not yet Fulfilled?. Cell Metab..

[98] Li J., Klughammer J., Farlik M., Penz T., Spittler A., Barbieux C., Berishvili E., Bock C., Kubicek S. (2016). Single-cell transcriptomes reveal characteristic features of human pancreatic islet cell types. EMBO Rep..

[99] Wang Y.J., Schug J., Won K.J., Liu C., Naji A., Avrahami D., Golson M.L., Kaestner K.H. (2016). Single-Cell Transcriptomics of the Human Endocrine Pancreas. Diabetes.

[100] Segerstolpe A., Palasantza A., Eliasson P., Andersson E.M., Andreasson A.C., Sun X., Picelli S., Sabirsh A., Clausen M., Bjursell M.K. (2016). Single-Cell Transcriptome Profiling of Human Pancreatic Islets in Health and Type 2 Diabetes. Cell Metab..

[101] Xin Y., Kim J., Okamoto H., Ni M., Wei Y., Adler C., Murphy A.J., Yancopoulos G.D., Lin C., Gromada J. (2016). RNA Sequencing of Single Human Islet Cells Reveals Type 2 Diabetes Genes. Cell Metab..

[102] Baron M., Veres A., Wolock S.L., Faust A.L., Gaujoux R., Vetere A., Ryu J.H., Wagner B.K., Shen-Orr S.S., Klein A.M. (2016). A Single-Cell Transcriptomic Map of the Human and Mouse Pancreas Reveals Inter- and Intra-cell Population Structure. Cell Syst..

[103] Muraro M.J., Dharmadhikari G., Grun D., Groen N., Dielen T., Jansen E., van Gurp L., Engelse M.A., Carlotti F., de Koning E.J. (2016). A Single-Cell Transcriptome Atlas of the Human Pancreas. Cell Syst..

[104] Camunas-Soler J., Dai X.Q., Hang Y., Bautista A., Lyon J., Suzuki K., Kim S.K., Quake S.R., MacDonald P.E. (2020). Patch-Seq Links Single-Cell Transcriptomes to Human Islet Dysfunction in Diabetes. Cell Metab..

[105] Gutierrez G.D., Gromada J., Sussel L. (2017). Heterogeneity of the Pancreatic Beta Cell. Front. Genet..

[106] Benninger R.K.P., Hodson D.J. (2018). New Understanding of beta-Cell Heterogeneity and In Situ Islet Function. Diabetes.

[107] Da Silva Xavier G., Rutter G.A. (2020). Metabolic and Functional Heterogeneity in Pancreatic beta Cells. J. Mol. Biol..

[108] Roscioni S.S., Migliorini A., Gegg M., Lickert H. (2016). Impact of islet architecture on beta-cell heterogeneity, plasticity and function. Nat. Rev. Endocrinol..

[109] Benninger R.K., Hutchens T., Head W.S., McCaughey M.J., Zhang M., Le Marchand S.J., Satin L.S., Piston D.W. (2014). Intrinsic islet heterogeneity and gap junction coupling determine spatiotemporal Ca^2+^ wave dynamics. Biophys. J..

[110] Serre-Beinier V., Le Gurun S., Belluardo N., Trovato-Salinaro A., Charollais A., Haefliger J.A., Condorelli D.F., Meda P. (2000). Cx36 preferentially connects beta-cells within pancreatic islets. Diabetes.

[111] Bavamian S., Klee P., Britan A., Populaire C., Caille D., Cancela J., Charollais A., Meda P. (2007). Islet-cell-to-cell communication as basis for normal insulin secretion. Diabetes Obes. Metab..

[112] Bosco D., Haefliger J.A., Meda P. (2011). Connexins: Key mediators of endocrine function. Physiol. Rev..

[113] Farnsworth N.L., Hemmati A., Pozzoli M., Benninger R.K. (2014). Fluorescence recovery after photobleaching reveals regulation and distribution of connexin36 gap junction coupling within mouse islets of Langerhans. J. Physiol..

[114] Gosak M., Markovic R., Dolensek J., Slak Rupnik M., Marhl M., Stozer A., Perc M. (2018). Network science of biological systems at different scales: A review. Phys. Life Rev..

[115] Hodson D.J., Schaeffer M., Romano N., Fontanaud P., Lafont C., Birkenstock J., Molino F., Christian H., Lockey J., Carmignac D. (2012). Existence of long-lasting experience-dependent plasticity in endocrine cell networks. Nat. Commun..

[116] Salem V., Silva L.D., Suba K., Georgiadou E., Neda Mousavy Gharavy S., Akhtar N., Martin-Alonso A., Gaboriau D.C.A., Rothery S.M., Stylianides T. (2019). Leader beta-cells coordinate Ca^2+^ dynamics across pancreatic islets in vivo. Nat. Metab..

[117] Westacott M.J., Ludin N.W.F., Benninger R.K.P. (2017). Spatially Organized beta-Cell Subpopulations Control Electrical Dynamics across Islets of Langerhans. Biophys. J..

[118] Hodson D.J., Mitchell R.K., Bellomo E.A., Sun G., Vinet L., Meda P., Li D., Li W.H., Bugliani M., Marchetti P. (2013). Lipotoxicity disrupts incretin-regulated human beta cell connectivity. J. Clin. Investig..

[119] Arrojo E.D.R., Jacob S., Garcia-Prieto C.F., Zheng X., Fukuda M., Nhu H.T.T., Stelmashenko O., Pecanha F.L.M., Rodriguez-Diaz R., Bushong E. (2019). Structural basis for delta cell paracrine regulation in pancreatic islets. Nat. Commun..

[120] Satin L.S., Zhang Q., Rorsman P. (2020). “Take Me To Your Leader”: An Electrophysiological Appraisal of the Role of Hub Cells in Pancreatic Islets. Diabetes.

[121] Satin L.S., Rorsman P. (2020). Response to Comment on Satin et al. “Take Me To Your Leader”: An Electrophysiological Appraisal of the Role of Hub Cells in Pancreatic Islets. Diabetes.

[122] Dwulet J.M., Briggs J.K., Benninger R.K.P. (2020). Small subpopulations of beta cells do not drive islet oscillatory [Ca^2+^] dynamics via gap junction communication. bioRxiv.

[123] Lu T.T.-H., Heyne S., Dror E., Casas E., Leonhardt L., Boenke T., Yang C.-H., Arrigoni L., Dalgaard K., Teperino R. (2018). The Polycomb-Dependent Epigenome Controls beta Cell Dysfunction, Dedifferentiation, and Diabetes. Cell Metab..

[124] El Hajj N., Schneider E., Lehnen H., Haaf T. (2014). Epigenetics and life-long consequences of an adverse nutritional and diabetic intrauterine environment. Reproduction.

[125] Soubry A., Schildkraut J.M., Murtha A., Wang F., Huang Z., Bernal A., Kurtzberg J., Jirtle R.L., Murphy S.K., Hoyo C. (2013). Paternal obesity is associated with IGF2 hypomethylation in newborns: Results from a Newborn Epigenetics Study (NEST) cohort. BMC Med..

[126] Soubry A., Murphy S.K., Wang F., Huang Z., Vidal A.C., Fuemmeler B.F., Kurtzberg J., Murtha A., Jirtle R.L., Schildkraut J.M. (2015). Newborns of obese parents have altered DNA methylation patterns at imprinted genes. Int. J. Obes. (Lond.).

[127] Feinberg A.P. (2007). Phenotypic plasticity and the epigenetics of human disease. Nature.

[128] Van de Pette M., Abbas A., Feytout A., McNamara G., Bruno L., To W.K., Dimond A., Sardini A., Webster Z., McGinty J. (2017). Visualizing Changes in Cdkn1c Expression Links Early-Life Adversity to Imprint Mis-regulation in Adults. Cell Rep..

[129] Van de Pette M., Galvao A., Millership S.J., To W.K., Dimond A., Prodani C., McNamara G., Bruno L., Sardini A., Webster Z. (2020). Epigenetic change induced by utero dietary challenge provokes phenotypic variability across multiple generations of mice. bioRxiv.

[130] Gluckman P.D., Hanson M.A., Buklijas T., Low F.M., Beedle A.S. (2009). Epigenetic mechanisms that underpin metabolic and cardiovascular diseases. Nat. Rev. Endocrinol..

[131] Jirtle R.L., Skinner M.K. (2007). Environmental epigenomics and disease susceptibility. Nat. Rev. Genet..

[132] Ng S.F., Lin R.C., Laybutt D.R., Barres R., Owens J.A., Morris M.J. (2010). Chronic high-fat diet in fathers programs beta-cell dysfunction in female rat offspring. Nature.

